# Morphometric study of normal acetabulum on computed tomography scan in adults at a tertiary care center

**DOI:** 10.1097/MS9.0000000000002394

**Published:** 2024-08-07

**Authors:** Bijay Adhikari, Pratik Adhikari, Manoj Bhattarai, Shashank Pokharel, Kapil Adhikari, Mukesh Kumar Gupta

**Affiliations:** B.P. Koirala Institute of Health Sciences, Dharan, Nepal

**Keywords:** acetabulum, age, computed tomography, morphometric parameters

## Abstract

**Background:**

The introduction provides an in-depth understanding of the acetabulum’s anatomical structure and its significance in hip joint biomechanics. It highlights the variability in acetabular morphology among normal individuals, emphasizing the importance of establishing normal ranges for accurate diagnosis of acetabular deformities. The section also underscores the role of geographical, racial, and anatomical factors in influencing acetabular parameters, crucial for orthopedic interventions and surgical outcomes like total hip arthroplasty. Furthermore, it discusses the limitations of traditional imaging methods and the necessity of advanced techniques like computed tomography (CT) scans for precise evaluation and preoperative planning in hip surgeries.

**Materials and methods:**

The study adopted a cross-sectional design at a tertiary care center, enrolling 94 participants using purposive sampling for CT evaluation of acetabular morphology. Morphometric parameters including center edge angle of Wiberg, acetabular angle of Sharp, acetabular version, acetabular depth, and joint space width were measured using CT scans, while BMI categorization and statistical analyses were conducted to explore gender-based and side-based differences and correlations with BMI and age.

**Results:**

The study included 94 patients with ages ranging from 20 to 82 years (mean age: 49±15 years), predominantly in the 41–50 years age group. Female participants slightly outnumbered males. The mean BMI was 23.5±3.2 kg/m^2^, with variations across different age and BMI categories. Most morphometric parameters showed no significant differences between sexes or sides but did correlate with age and BMI, notably the center edge angle positively correlating with BMI in males.

**Conclusions:**

The study found correlations between morphometric parameters of the acetabulum and both age and BMI. Specifically, the center edge angle demonstrated a positive correlation with BMI in males and a weak positive correlation with age. Acetabular angle showed a negative correlation with BMI, highlighting potential implications for understanding hip joint health and conditions related to BMI changes.

## Introduction

Highlights

- Our study’s precise morphometric measurements of the normal acetabulum using computed tomography (CT) scans aid in distinguishing hip joint conditions, reducing misdiagnoses, and guiding accurate surgical interventions, crucial for optimizing outcomes such as reducing femoroacetabular impingement risks.
- By correlating acetabular morphology with age and BMI across diverse demographics, our research offers tailored treatment insights in orthopedic care, ensuring personalized interventions and enhancing patient satisfaction and recovery.
- Aligned with global research, our findings contribute comparative insights into acetabular morphometry, crucial for developing standardized guidelines worldwide and improving clinical practices, particularly impactful in resource-limited healthcare contexts like Nepal.
- Through detailed assessment of acetabular parameters, our study aids in preoperative planning for hip reconstructive surgeries, reducing risks such as dislocations post-surgery, emphasizing the clinical importance of accurate diagnostic tools and surgical guidance in orthopedics.

The acetabulum, a key component of the hip joint, is a concave cavity formed by the fusion of three bones: the ilium, ischium, and pubis. It plays a crucial role in maintaining the stability and functionality of the hip joint by providing a socket for articulation with the femoral head^1^. Variations in acetabular morphology are common even among individuals considered anatomically normal, making it imperative to establish a baseline understanding of acetabular parameters. This knowledge aids in distinguishing between normal anatomical variation and pathological deformities, facilitating accurate diagnosis and treatment planning^2^.

ChatGPT is more successful than students in anatomy education and has the potential to increase student participation but may make errors in basic anatomy knowledge^3^.

Geographic trends and ethnic differences influence acetabular morphology, with genetic factors being the primary determinant^4^. However, cultural, environmental, and social factors may also contribute to variations in hip development. Understanding these variations is essential for radiologists, orthopedicians, and prosthetists alike, as it informs clinical decision-making and prosthetic implant design^5,6^. Moreover, abnormalities in acetabular morphology can lead to biomechanical alterations, predisposing individuals to conditions such as femoroacetabular impingement and osteoarthritis of the hip^7^.

Accurate assessment of acetabular orientation is paramount in hip reconstructive surgery to prevent postoperative complications such as dislocation and implant malposition^8^. Malpositioning of the acetabular component can result in increased wear, instability, and revision surgeries, underscoring the importance of precise surgical techniques^9^. Morphometric studies play a crucial role in establishing the normal range of acetabular parameters and identifying deviations from the norm^10^. Three-dimensional computed tomography (3D-CT) reconstructions and anatomical models enhance veterinary anatomy education by providing detailed, realistic views of anatomical structures, aiding in diagnosis and treatment of anatomical disorders^11^. While plain radiographs are commonly used for initial assessment, they may lack the sensitivity and specificity required for comprehensive evaluation^12^.

Advancements in imaging modalities, particularly multidetector CT (MDCT), have revolutionized the assessment of acetabular morphology^13^. MDCT offers superior visualization and multiplanar evaluation, facilitating accurate preoperative planning and implant placement. By overcoming the limitations of plain radiography, MDCT has become the imaging modality of choice for evaluating acetabular morphology in patients undergoing hip surgery^14^. This study aims to investigate normal acetabular morphometric measurements in adults, with implications for early pathological diagnosis and improved prosthetic implant design, particularly in the context of Nepal where such data are scarce^15^.

## Materials and methods

### Study design, sample size and sampling technique

We carried out a cross-sectional at a tertiary care center, over a period of 1 year. According to the study by Saikia *et al*.^16^ in 2008, the mean acetabular angle of Sharp was found to 39.2° with a standard deviation of 4.9°. To determine the sample size needed for this study with a relative precision of 2% and a 95% CI, we calculated the sample size using the sample mean form.

Given:

Mean acetabular angle of Sharp (A.D. Mean)=39.2°

Standard deviation (S.D.)=4.9°

Relative precision (L)=2% of mean=0.98°

Z value for 95% CI=1.95


$$n=z^{2}x\sigma^{2}L2$$

Where Z=1.95 at 95% CI σ=4.9


$$L=2.5\%\;\mathrm{ofmean}=0.98n=1.95^{2}x4.9^{2}/0.98^{2}=94$$

Therefore, the calculated sample size required for our study is 94 participants.

A purposive sampling method was adopted for the collection of the study samples. The study included persons undergoing CT scan of abdominal and pelvis for various clinical indications in whom hip joint was optimally visualized. The study excluded individuals aged 19 years or younger, those with a hip injury, individuals with a hip joint implant or prosthesis, and those in whom hip abnormality was incidentally detected on a CT scan. The work has been carried out in accordance with the STROCCS criteria^17^.

### Measurement of acetabulum and its area

Morphometric study of normal acetabulum was done using a 16-slice CT scanner machine available in the tertiary care center. CT was done in supine position and CT protocol was based on clinical indications of the patient as employed in the department. Following CT, multiplanar reformation were obtained. The measurement of CE angle of Wiberg, acetabular depth and joint space width were done on coronal reformatted images, whereas acetabular angle of sharp was measured on frontal projection scanogram and acetabular version was measured on axial section.

**Figure 1:**
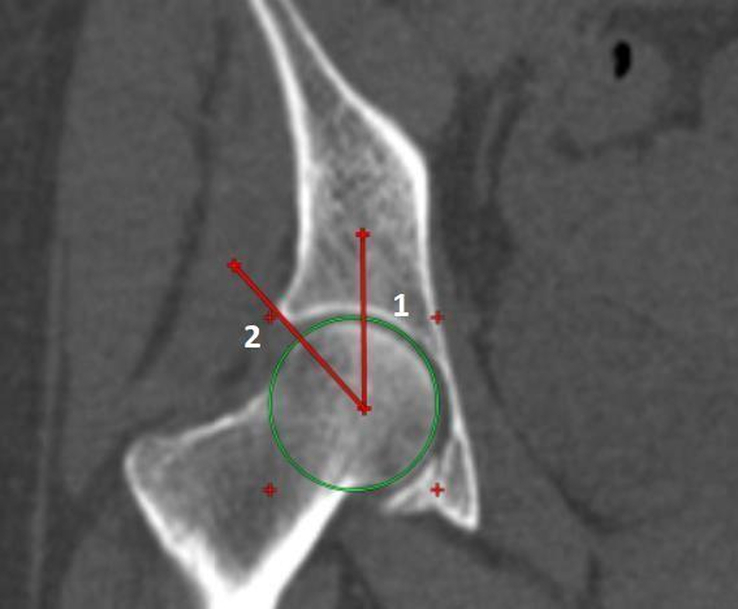
Reformatted coronal computed tomography image of right hip joint showing method for measurement of center edge angle of Wiberg (Line 1: Vertical line drawn through center of femoral head, Line 2: Line drawn from center of femoral head to lateral margin of acetabulum: Center edge angle: Angle between line 1 and 2).

**Figure 2:**
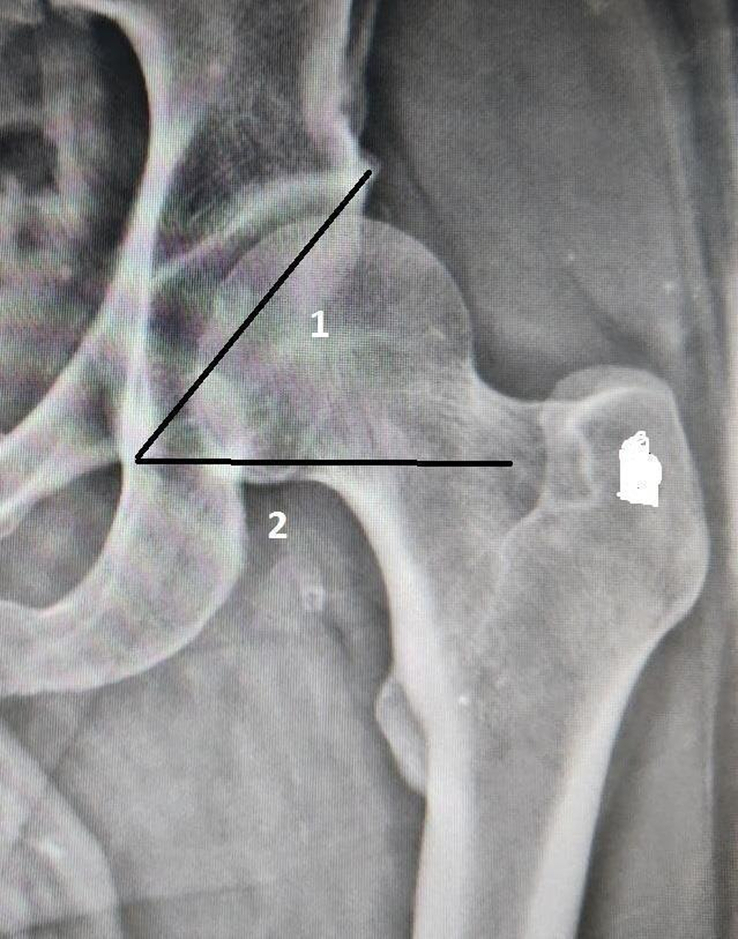
Frontal scanogram image of left hip showing method for measurement of acetabular angle of Sharp (Line 1: Line joining tip of tear drop to lateral margin of acetabulum, Line 2: Line drawn perpendicular to the tip of tear drop: Acetabular angle: Angle between line 1 and 2).

**Figure 3:**
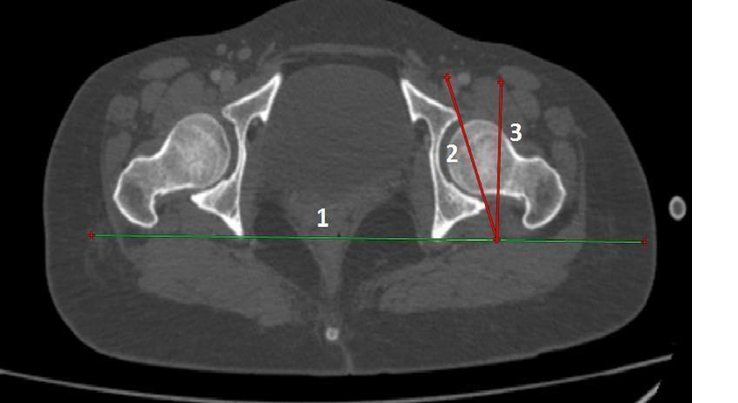
Axial computed tomography image of bilateral hip joint showing method for measurement of acetabular version (Line 1: Line connecting bilateral posterior ischia, Line 2: Line connecting anterior and posterior acetabular margins, Line 3: Line drawn perpendicular to line 1 from point of intersection of line 1 and 2: Acetabular version: Angle between line 2 and 3).

**Figure 4:**
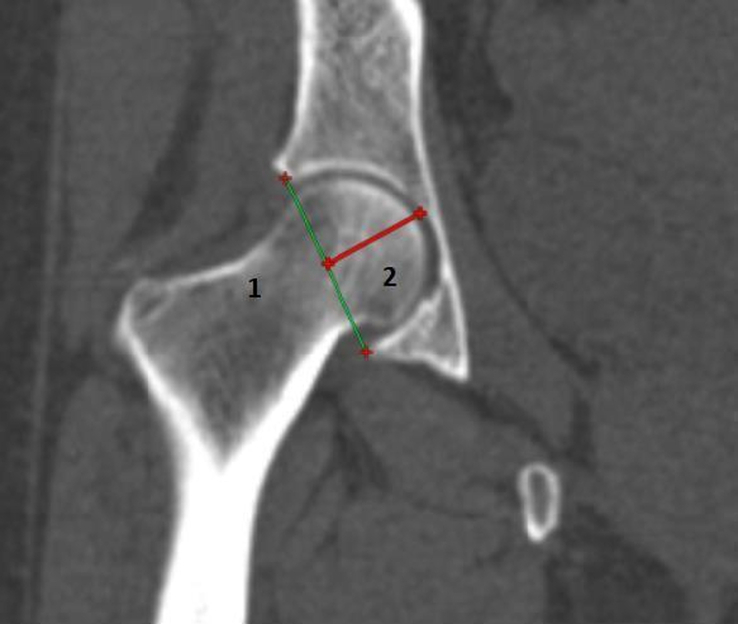
Reformatted coronal computed tomography image of right hip joint showing method for measurement of acetabular depth (Line 1: Line connecting superior and inferior acetabular margins, Line 2: Acetabular depth (perpendicular line drawn from midpoint of line 1 to dome of acetabulum).

**Figure 5:**
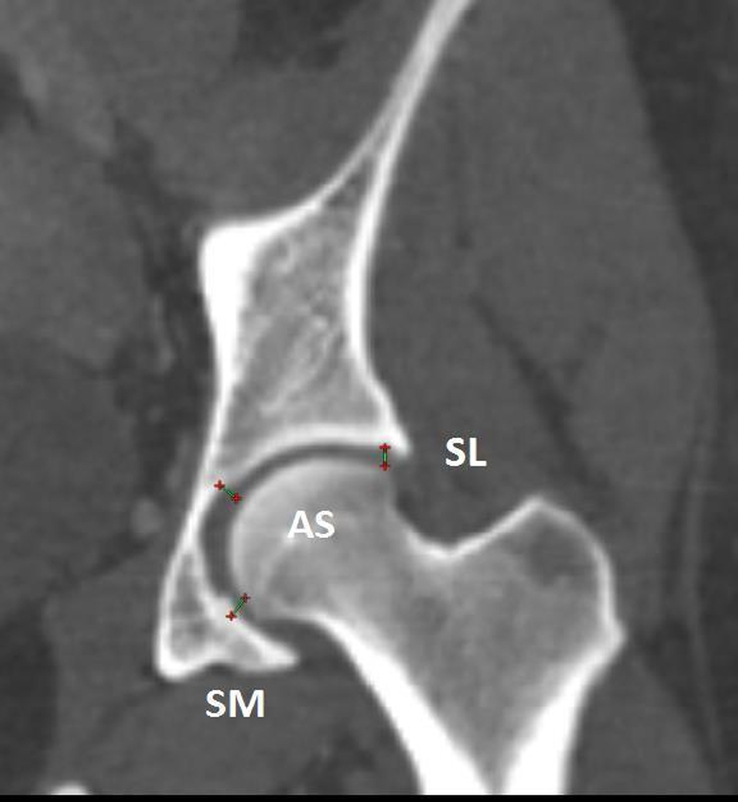
Reformatted coronal computed tomography image of left hip joint showing method for measurement of joint space width. AS, joint space width at apical site; SL, joint space width at superolateral site; SM, joint space width at superomedial site.

Definition of parameters used are as follows:

- CE angle of Wiberg (Fig. 1): The angle between a line drawn vertically through the center of the femoral head and a line drawn from the center of the femoral head to the lateral margin of the acetabulum^7,18,19^.
- Acetabular angle of Sharp (Fig. 2): The angle between the horizontal line drawn through the tip of pelvic tear drop and a line from the tip of the tear drop to the lateral margin of the acetabulum^7,18^.
- Acetabular version (Fig. 3): The angle formed by the intersection of line connecting the posterior and anterior margins of the acetabulum with a line drawn perpendicular to the line connecting the bilateral posterior ischia^18^.
- Acetabular depth (Fig. 4): The perpendicular distance from the mid- point of a line connecting the superior and the inferior acetabular margins up to the dome of the acetabulum^7,18^.
- Joint space width (Fig. 5): The joint space width of the hip refers to the interbone area between the acetabular roof and the part of the femoral head facing it. The measurements were taken at three different places

(a) Superolateral site (SL), (b) Apical site (AS), (c) Superomedial site (SM body weight and height of all the participants were measured and BMI was calculated. BMI is defined as a person’s weight in kg/height in m^2^ and on the basis of the participants were categorized as^20^:

Underweight <18.5 kg/m^2^

Normal range 18.5–24.9 kg/m^2^

Overweight 25.0–29.9 kg/m^2^

### Data entry and analysis

The data collected was tabulated in Microsoft Excel and analysis was carried out using Statistical Package for Social Sciences (SPSS) version 11.5. Mean±SD was calculated for center edge (CE) angle of Wiberg, acetabular angle of sharp (As), acetabular version (Av), acetabular depth (Ad) and joint space width (JSW). For comparing the mean values of morphometric parameters according to body side (left vs. right) paired samples *t*-test was used. Independent sample *t*-test was used to compare the means of morphometric parameters in male and female. Pearson’s correlation test was used to see correlation of above-mentioned morphometric parameters with BMI and age. *P* less than 0.05 was considered as significant.

## Results

A total of 94 patients, meeting the selection criteria were included in this study.

Age of patients ranged from 20 to 82 years (mean age: 49±15 years) with maximum number of cases, 22 (23.4%) in 41–50 years of age group as shown in Table 1. Mean age of males and females was 48.3 ±16.4 years and 50.5±13.7 years, respectively.

**Table 1:** Distribution of patients according to age.

Age (year)	Frequency (*n*=94) (percent), *n* (%)
20–30	11 (11.7)
31–40	17 (18.1)
41–50	22 (23.4)
51–60	19 (20.2)
61–70	13 (13.8)
>70	12 (12.7)

**Figure 6:**
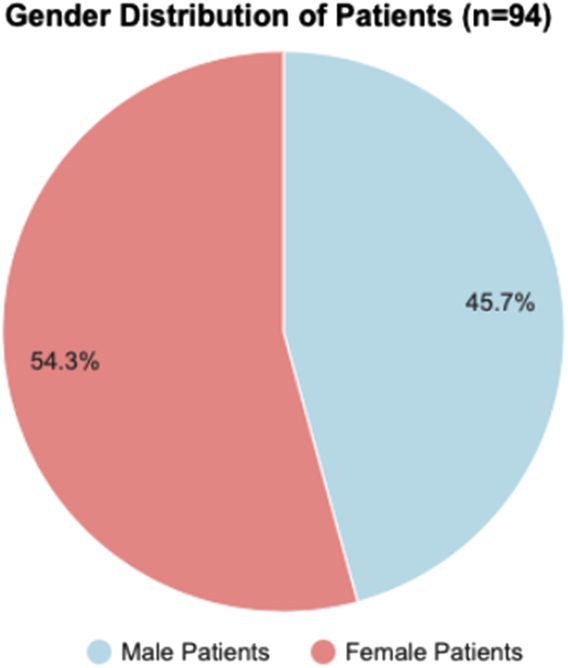
Pie chart showing distribution of patients according to sex.

Out of a total of 94 patients, 43 (45.7%) were males and 51 (54.3%) females, with male and female ratio of 0.79:1 as shown in Fig. 6.

**Figure 7:**
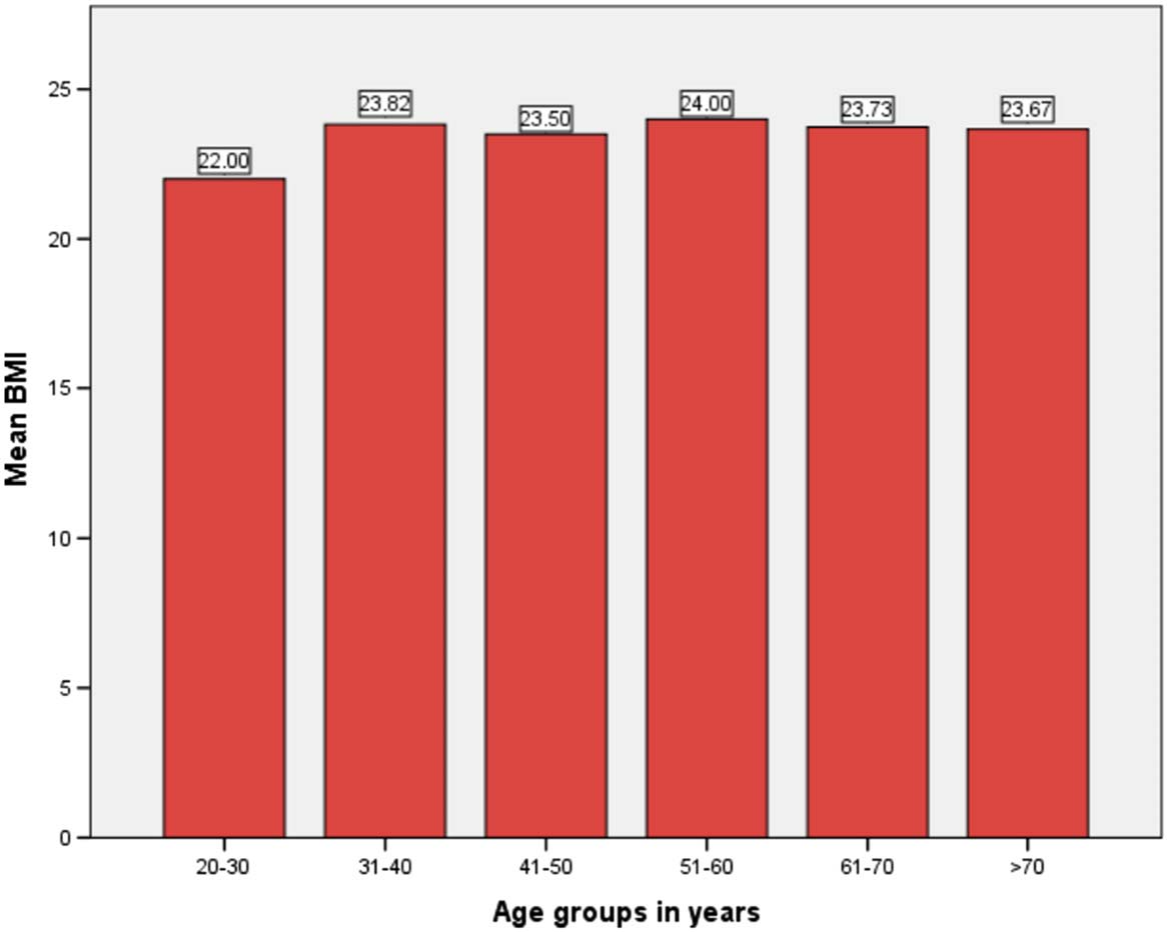
Bar diagram showing mean BMI in different age groups.

BMI of participants ranged from 16.00–31.00 (mean BMI: 23.5±3.2) kg/m^2^ BMI in male ranged from 16.00 to 31.00 (mean BMI: 23.28±3.3) and mean BMI in females ranged from 16.00 to 30.00 (mean BMI: 23.76±3.2) kg/m^2^ as shown in Fig. 7.

The mean Central edge angle, Acetabular angle, Acetabular version and Acetabular depth were 45.07±4.92°, 35.12±4.17°, 22.00±3.48° and 18.01±2.07 mm, respectively. The mean hip joint space width at superomedial (SM), apical site (AS) and superolateral (SL) site were 7.04±1.22, 6.70±1.05 and 5.27±1.17 mm, respectively. (Table 2)

**Table 2:** Mean±SD and range of various morphometric parameters of acetabulum (*n*=94).

Parameters	Mean±SD (*n*=94)	Range
Center edge angle (°)	45.07±4.92	27.0–58.0
Acetabular angle (°)	35.12±4.17	25.5–46.5
Acetabular version (°)	22.00±3.48	14.5–35.5
Acetabular depth (mm)	18.01±2.07	12.0–24.0
Joint space width SM (mm)	7.04±1.22	4.5–11.5
Joint space width AS (mm)	6.70±1.05	4.5–9.0
Joint space width SL (mm)	5.27±1.17	2.0–7.5

AS, apical site; SL, superolateral site; SM, superomedial site.

Mean values of CE angle ranged from 40.1±5.8 to 46.8±3.6° with maximum value seen in the 51–60 years of age group. Similarly mean values of acetabular angle ranged from 33.9±4.01 to 37 ±5.9° with the maximum value seen in 51–60 years of age group. Mean values of acetabular version ranged from 19.9±2.1 to 23.6±2.3° with maximum value observed in older than 70 years of age group. The mean value of acetabular depth ranged from 17.4±1.9 to 18.4±3.1 mm with the maximum value observed in older than 70 years of age group. The mean value of joint space width at SM, AS and SL sites ranged from 6.7±1.1 to 7.5±1.4 mm, 6.3±1.1 to 6.9±1 mm and 4.5±1.1 to 5.6±1.2 mm, respectively, with highest value seen in 20–30 years of age group in joint space width at SM, AS and sites in age group 61–70 years in joint space width at SL site. (Table 3)

**Table 3:** Mean±SD of various topographic measurements in different age groups (*n*=94).

Age (year)	Center edge angle (°)	Acetabular angle (°)	Acetabular version (°)	Acetabular depth (mm)	Joint space width SM (mm)	Joint space width AS (mm)	Joint space width SL (mm)
20–30 (*n*=11)	40.1±5.8	37±5.9	19.9±2.1	17.4±1.9	7.5±1.4	6.9±1.0	5.6 ±0.9
31–40 (*n*=17)	45.7±4.6	35.7±3.6	22.4±4.2	17.8±2	7.3±1.5	6.7±0.8	4.55±1.1
41–50 (*n*=22)	45.1±3.7	34.5±3.7	22.2±4.19	18±1.6	6.9±1.0	6.8±1.0	5.6±1.1
51–60 (*n*=19)	46.8±4.8	33.9±4.01	21.6±2.9	18.1±2.3	6.7±1.0	6.5±1.1	5.1±0.8
61–70 (*n*=13)	46.3±3.6	34.7±4.1	22.1±3.1	17.8±1.6	6.8±0.8	6.3±1.1	5.6±1.2
>70 (*n*=12)	43.7±5.94	36.3±3.90	23.6±2.3	18.4±3.1	7.2±1.3	6.8±1.1	5.0±1.4

AS, apical site; SL, superolateral site; SM, superomedial site.

Mean values of CE angle ranged from 39.2±3.2 to 49.2±5.7° with maximum value seen in obese BMI group. Similarly mean values of acetabular angle ranged from 33.5±4.6 to 35.5 ±3.5° with maximum value seen in normal BMI group. Mean values of acetabular version ranged from 20.7±1.7 to 23.1±2.3° with maximum value observed in normal BMI group. Mean value of acetabular depth ranged from 17.6±1.4 to 19.5±2.5 mm with maximum value observed in obese BMI group. Mean value of joint space width at SM, AS and SL sites ranged from 6.6±0.8 to 7.6±1.1 mm, 6.5±1.0 to 6.9±0.9 mm and 4.6±0.9 to 5.4±1.5 mm, respectively, with maximum value observed in underweight BMI group category (Table 4).

**Table 4:** Mean±SD of various topographic measurements in different BMI groups (*n*=94).

BMI categories	Center edge angle (°)	Acetabular angle (°)	Acetabular version (°)	Acetabular depth (mm)	Joint space width SM (mm)	Joint space width AS (mm)	Joint space width SL (mm)
Underweight (<18.5) (*n*=5)	39.2±3.2	35.5±3.5	23.1±2.3	17.9±3.6	7.6±1.1	6.9±0.9	5.4±1.5
Normal (18.5–24.9) (*n*=56)	44.9±4.8	35.8±4.2	22.0±3.7	18.0±2.0	7.1±1.3	6.7±1.0	5.3±1.2
Overweight (25–29.9) (*n*=26)	45.2±4.0	33.8±3.8	22.0±3.4	17.6±1.4	6.6±0.8	6.5±1.0	5.3±0.9
Obese (>30) (*n*=7)	49.2±5.7	33.5±4.6	20.7±1.7	19.5±2.5	7.0±1.1	6.6±0.8	4.6±0.9

AS, apical site; SL, superolateral site; SM, superomedial site.

Mean±SD for each of the measured parameters were calculated for male and females respectively. Mean values of CE angle, acetabular angle, acetabular version was slightly higher in females as compared to male but the differences were statistically insignificant. Acetabular depth, joint space width at SM, AS and SL sites were found to be higher in male as compared to females but the differences were statistically insignificant (Table 5).

**Table 5:** Comparison of mean±SD of various topographic measurements in male and female (*n*=94).

Parameters	Male (*n*=43)	Female (*n*=51)	^a^ *P*
Center edge angle (°)	44.5±5.5	45.4±4.3	0.371
Acetabular angle (°)	34.3±3.9	35.7±4.2	0.121
Acetabular version (°)	21.4±2.3	22.5±4.1	0.137
Acetabular depth (mm)	18.4±2.3	17.6±1.7	0.081
Joint space width SM (mm)	7.2±1.3	6.8±1.0	0.311
Joint space width AS (mm)	6.7±1.0	6.6±1.0	0.582
Joint space width SL (mm)	5.4±1.2	5.1±1.1	0.183

AS, apical site; SL, superolateral site; SM, superomedial site.

^a^ Independent *t*-test.

Mean±SD for each of the measured parameters were calculated for right and left sides, respectively. Mean values of CE angle, acetabular version and joint space width at SL were higher on the right side as compared to left but the differences were statistically insignificant. Acetabular angle, acetabular depth, joint space width at SM and AS sites was higher on left side as compared to right but the differences were statistically insignificant (Table 6).

**Table 6:** Comparison of mean±SD of various topographic measurements on right and left side.

Topographic measurements	Side	Mean±SD	^a^ *P*
Center edge angle	Right	45.37±5.72	0.172
	Left	44.78±4.95	
Acetabular angle	Right	34.98±4.76	0.385
	Left	35.27±4.16	
Acetabular version	Right	22.2±3.81	0.151
	Left	21.8±3.65	
Acetabular depth	Right	17.95±2.61	0.613
	Left	18.07±2.17	
Joint space width SM	Right	6.96±1.58	0.354
	Left	7.14±1.50	
Joint space width AS	Right	6.56±1.47	0.192
	Left	6.84±1.45	
Joint space width SL	Right	5.31±1.47	0.657
	Left	5.23±1.37	

AS, apical site; SL, superolateral site; SM, superomedial site.

^a^ Paired *t*-test.

On comparison of mean values of various topographic measurements with sex, no significant difference was obtained. The CE angle, acetabular angle and acetabular version were higher in females as compared to male on both right and left sides. Acetabular depth, Joint space width SL, AS and SM were higher in male as compared to female (Table 7).

**Table 7:** Comparison of mean of various topographic measurements in male and female on right and left side.

Topographic measurements	Sex	*N*	Mean±SD	^a^ *P*
Center edge angle right	Male	43	44.60±6.02	0.235
	Female	51	46.02±5.43	
Center edge angle left	Male	43	44.56±5.55	0.697
	Female	51	44.96±4.47	
Acetabular angle right	Male	43	34.35±4.68	0.241
	Female	51	35.51±4.81	
Acetabular angle left	Male	43	34.37±3.74	0.056
	Female	51	36.02±4.37	
Acetabular version right	Male	43	21.74±2.96	0.288
	Female	51	22.59±4.40	
Acetabular version left	Male	43	21.07±2.43	0.076
	Female	51	22.41±4.36	
Acetabular depth right	Male	43	18.35±2.85	0.17
	Female	51	17.61±2.42	
Acetabular depth left	Male	43	18.49±2.37	0.09
	Female	51	17.73±1.94	
Joint space width SL right	Male	43	7.26±1.77	0.094
	Female	51	6.71±1.37	
Joint space width SL left	Male	43	7.21±1.68	0.677
	Female	51	7.08±1.35	
Joint space width AS right	Male	43	6.60±1.51	0.807
	Female	51	6.53±1.46	
Joint space width AS left	Male	43	6.93±1.42	0.585
	Female	51	6.76±1.49	
Joint space width SM right	Male	43	5.47±1.59	0.347
	Female	51	5.18±1.36	
Joint space width SM left	Male	43	5.35±1.41	0.459
	Female	51	5.14±1.34	

AS, apical site; SL, superolateral site; SM, superomedial site.

^a^ Independent *t*-test.

Pearson correlation test was applied to determine the correlation between various topographic measurements with age. The CE angle, acetabular version, acetabular depth, joint space width at SM site showed statistically insignificant weak positive correlation. Acetabular angle, joint space width at AS and SL sites showed statistically insignificant negative correlation with age (Table 8).

**Table 8:** Correlation of various topographic measurements with age (*n*=94).

Parameter	^a^ r value	*P*
Center edge angle	0.17	0.12
Acetabular angle	−0.91	0.38
Acetabular version	0.15	0.13
Acetabular depth	0.13	0.18
Joint space width SM	0.01	0.85
Joint space width AS	−0.08	0.42
Joint space width SL	−0.10	0.30

AS, apical site; SL, superolateral site; SM, superomedial site.

^a^ Pearson correlation coefficient.

Pearson correlation test was applied to determine the correlation between the various topographic measurements with age. The CE angle, acetabular version, acetabular depth of both sides and joint space at the SL site on the right side showed statistically insignificant weak positive correlation with age. Acetabular angle, joint space width at SM, joint space width at AS on both sides and joint space width SL on left side showed statistically insignificant negative correlation with age (Table 9).

**Table 9:** Correlation of various topographic measurements on right and left side with age (*n*=94).

Topographic measurements	Side	^a^r value	*P*
Center edge angle	Right	0.19	0.05
	Left	0.11	0.28
Acetabular angle	Right	−0.08	0.44
	Left	−0.09	0.37
Acetabular version	Right	0.16	0.12
	Left	0.12	0.21
Acetabular depth	Right	0.11	0.26
	Left	0.12	0.24
Joint space width SM	Right	−0.14	0.16
	Left	−0.02	0.83
Joint space width AS	Right	−0.02	0.81
	Left	−0.14	0.16
Joint space width SL	Right	0.03	0.76
	Left	−0.02	0.98

AS, apical site; SL, superolateral site; SM, superomedial site.

^a^ Pearson correlation coefficient.

**Figure 8:**
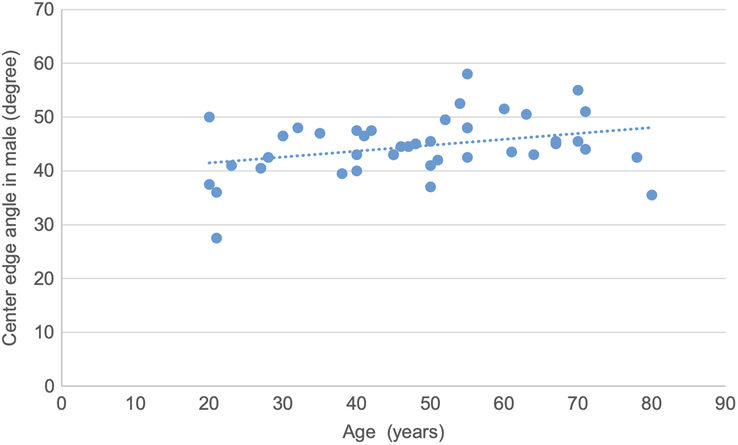
Scatter plot showing weak positive correlation of center edge angle with age in male participants (r=0.32, *P*=0.03).

A scatter plot is drawn showing a weak positive correlation between CE angle and age in male participants, with a correlation coefficient of 0.32 and a *P* value of 0.03 (Fig. 8).

**Figure 9:**
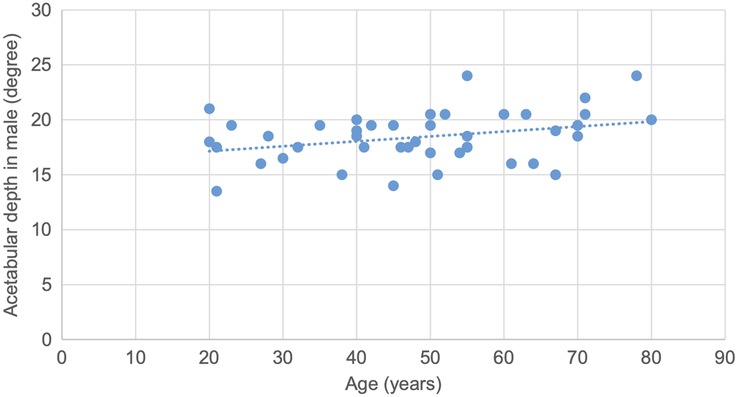
Scatter plot showing weak positive correlation of acetabular depth with age in male (r=0.31, *P*=0.03).

A scatter plot is drawn showing a weak positive correlation between acetabular depth and age in male participants, with a correlation coefficient of 0.31 and a *P* value of 0.03 (Fig. 9).

Pearson correlation test was used to determine the correlation of various topographic measurements in male and female with age. The CE angle and acetabular depth in male showed statistically significant weak positive correlation with age. The CE angle in female, acetabular angle in male, acetabular depth in female, joint space width at SM site in female, joint space width at AS and SL sites in both male and female showed statistically insignificant negative correlation. Acetabular angle in female, acetabular version in male and female and joint space width at SM site in male showed statistically insignificant weak positive correlation. (Figs. 8, 9 and Table 10).

**Table 10:** Correlation of various topographic measurements in male and female with age (*n*=94).

Parameter	Side	^a^r value	*P*
Center edge angle	Male	0.32	0.03
	Female	−0.04	0.78
Acetabular angle	Male	−0.26	0.09
	Female	0.03	0.79
Acetabular version	Male	0.12	0.44
	Female	0.17	0.21
Acetabular depth	Male	0.31	0.03
	Female	−0.06	0.67
Joint space width SM	Male	0.07	0.63
	Female	−0.02	0.84
Joint space width AS	Male	−0.08	0.57
	Female	−0.06	0.63
Joint space width SL	Male	−0.05	0.71
	Female	−0.14	0.29

AS, apical site; SL, superolateral site; SM, superomedial site.

^a^ Pearson correlation coefficient.

**Figure 10:**
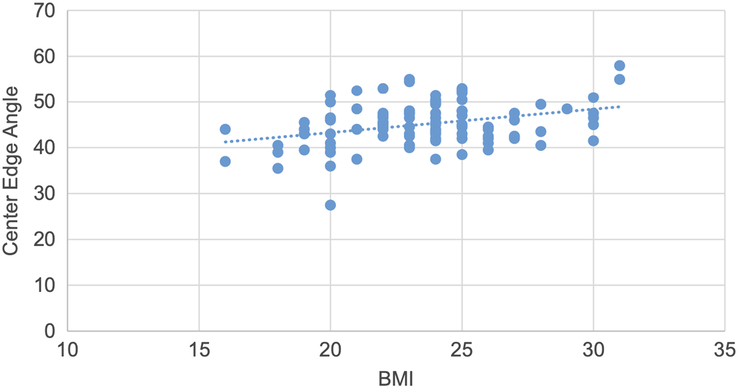
Scatter plot showing weak positive correlation of center edge angle with BMI (r=0.33, *P*=0.01).

A scatter plot is drawn showing a weak positive correlation between CE angle and BMI, with a correlation coefficient of 0.33 and a *P* value of 0.01 (Fig. 10).

**Figure 11:**
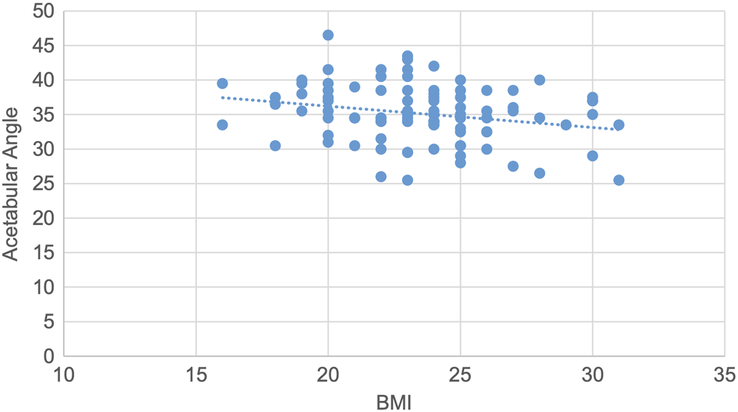
Scatter plot showing negative correlation of acetabular angle with BMI (r=−0.24, *P*=0.02).

A scatter plot is drawn showing a negative correlation between acetabular angle and BMI, with a correlation coefficient of −0.24 and a *P* value of 0.02 (Fig. 11).

Pearson correlation test was used to determine the correlation of various topographic measurements with BMI. The CE angle showed statistically significant weak positive correlation. The acetabular angle showed statistically significant negative correlation. Acetabular version, joint space width at SM, AS and SL sites showed statistically insignificant negative correlation. Acetabular depth showed statistically insignificant weak positive correlation. (Figs. 10,11 and Table 11).

**Table 11:** Correlation of various topographic measurements with BMI (*n*=94).

Parameter	^a^r value	*P*
Center edge angle	0.33	0.01
Acetabular angle	−0.24	0.02
Acetabular version	−0.04	0.64
Acetabular depth	0.07	0.49
Joint space width SM	−0.08	0.41
Joint space width AS	−0.09	0.37
Joint space width SL	−0.13	0.18

AS, apical site; SL, superolateral site; SM, superomedial site.

^a^ Pearson correlation coefficient.

**Figure 12:**
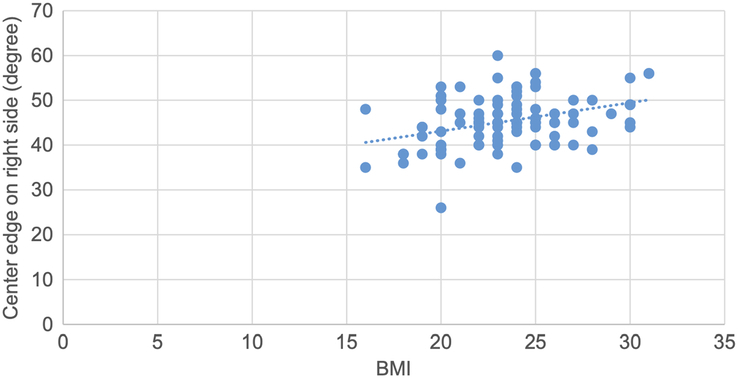
Scatter plot showing weak positive correlation of center edge angle with BMI on right side (r=0.35, *P*=0.01).

A scatter plot is drawn showing a weak positive correlation between the CE angle and BMI on the right side, with a correlation coefficient of 0.35 and a *P* value of 0.01 (Fig. 12).

**Figure 13:**
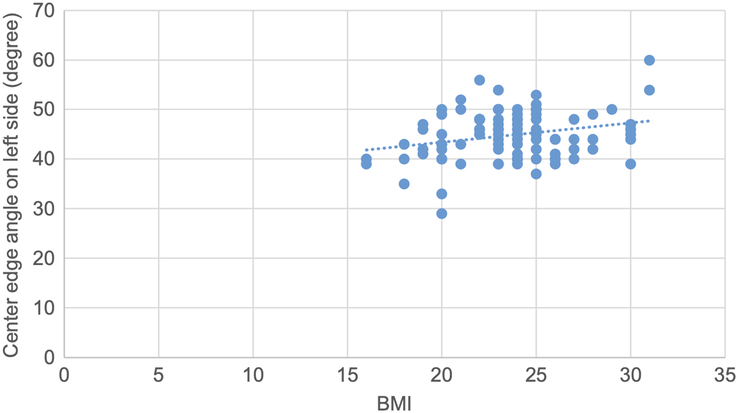
Scatter plot showing weak positive correlation of center edge angle with BMI on left side (r=0.25, *P*=0.01).

A scatter plot is drawn showing a weak positive correlation between CE angle and age, with a correlation coefficient of 0.32 and a *P* value of 0.03 (Fig. 13).

**Figure 14:**
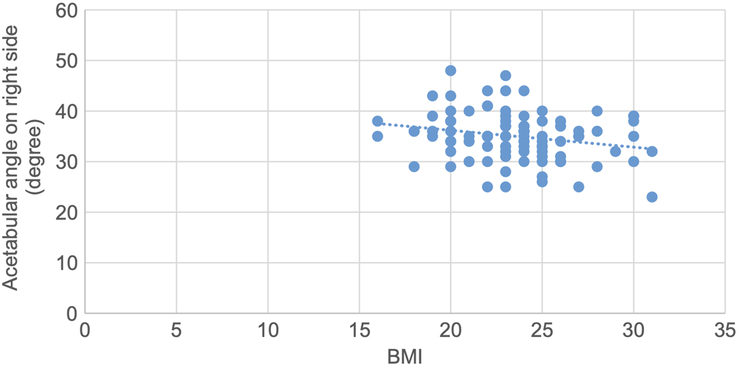
Scatter plot showing negative correlation of acetabular angle with BMI on right side (r=−0.22, *P*=0.02).

A scatter plot is drawn showing a negative correlation between acetabular angle and BMI on the right side, with a correlation coefficient of −0.22 and a *P* value of 0.02 (Fig. 14).

**Figure 15:**
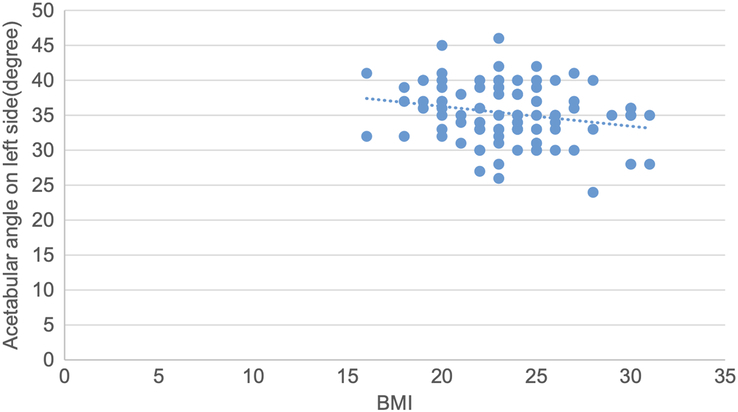
Scatter plot showing negative correlation of acetabular angle with BMI on left side (r=−0.22, *P*=0.03).

A scatter plot is drawn showing a negative correlation between acetabular angle and BMI on the left side, with a correlation coefficient of −0.22 and a *P* value of 0.03 (Fig. 15).

Pearson correlation coefficient test was used to determine the correlation between various topographic measurements with BMI and *P* values were obtained. The CE angle on both sides showed statistically significant weak positive correlation with BMI. Acetabular angle on both sides showed statistically significant negative correlation with BMI. The acetabular version on both sides, acetabular depth on left side, joint space width SM on both sides, joint space width AS on left side and joint space width SL on right side showed statistically insignificant negative correlation with BMI. Acetabular depth on right, joint space width AS on right side and joint space width SL on left side showed statistically insignificant weak positive correlation with BMI (Figs. 12, 13, 14, 15 and Table 12).

**Table 12:** Correlation of various topographic measurements in right and left side with BMI (*n*=94).

Topographic measurements	Side	^a^r value	*P*
Center edge angle	Right	0.35	0.01
	Left	0.25	0.01
Acetabular angle	Right	−0.22	0.02
	Left	−0.22	0.03
Acetabular version	Right	−0.06	0.51
	Left	−0.02	0.85
Acetabular depth	Right	0.11	0.26
	Left	−0.06	0.95
Joint space width SM	Right	−0.18	0.07
	Left	−0.03	0.76
Joint space width AS	Right	0.08	0.43
	Left	−0.05	0.63
Joint space width SL	Right	−0.16	0.11
	Left	0.02	0.78

AS, apical site; SL, superolateral site; SM, superomedial site.

^a^ Pearson correlation coefficient.

**Figure 16:**
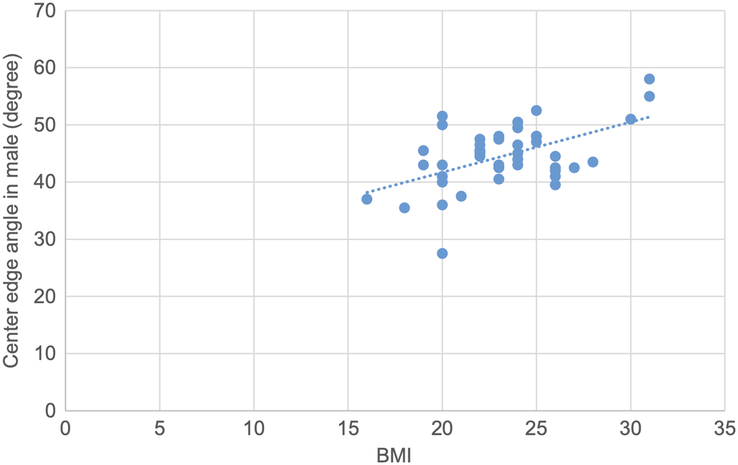
Scatter plot showing moderate positive correlation of center edge angle with BMI in male (r=−0.56, *P*=0.01).

**Figure 17:**
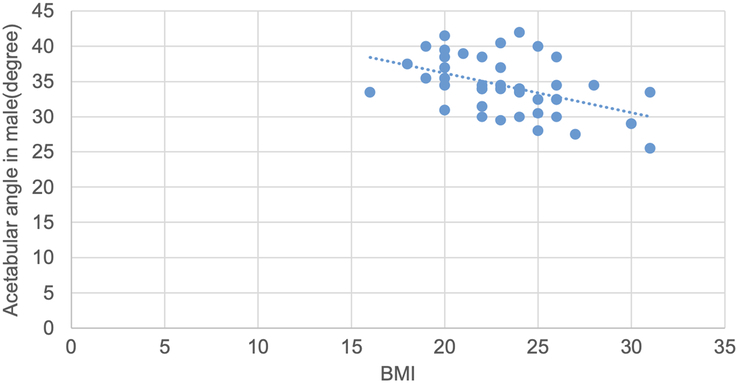
Scatter plot showing negative correlation of acetabular angle with BMI in male (r=−0.46, *P*=0.02).

Pearson correlation test was used to determine the correlation of various topographic measurements in male and female with BMI. The CE angle in male showed statistically significant moderate strong positive correlation. The acetabular angle showed statistically significant negative correlation. CE angle in female, acetabular depth in male and female and joint space width SL in female showed statistically insignificant weak positive correlation. Acetabular angle in female, acetabular version in male and female, joint space width at SM, AS sites in both male and female and joint space width at SL site in male showed statistically insignificant negative correlation (Figs. 16, 17 and Table 13).

**Table 13:** Correlation of various topographic measurements in male and female with BMI (*n*=94).

Parameter	Side	r	*P*
Center edge angle	Male	0.52	0.01
	Female	0.12	0.37
Acetabular angle	Male	−0.46	0.02
	Female	−0.09	0.53
Acetabular version	Male	−0.05	0.73
	Female	−0.06	0.63
Acetabular depth	Male	0.01	0.91
	Female	0.16	0.24
Joint space width SM	Male	−0.19	0.22
	Female	−0.03	0.83
Joint space width AS	Male	−0.15	0.32
	Female	−0.02	0.84
Joint space width SL	Male	−0.25	0.09
	Female	0.01	0.94

AS, apical site; SL, superolateral site; SM, superomedial site.

* Pearson correlation coefficient.

## Discussion

Various authors have introduced radiographic parameters of the acetabulum on plain radiographs, with techniques to measure these parameters subsequently evaluated by several studies^2,16,21,22^. Plain radiographs, despite being the initial imaging investigation, have limitations because they assess only a focal region of the acetabular rim, thus failing to adequately identify and quantify the severity of acetabular deformity. Consequently, their reliability in diagnosing dysplasia or impingement morphology, even among experienced radiologists, is questionable.

Our study evaluated 94 patients and 188 normal acetabula, with a demographic breakdown of 43 males (45.7%) and 51 females (54.3%), ranging in age from 20 to 82 years, with a mean age of 49±15 years. Most patients fell within the 41–50 years age group, and the mean BMI ranged from 16 to 31 kg/m², with a maximum BMI in the 51–60 years age group (24 kg/m²). This aligns with a study by Saikia *et al.* ^16^ on a North East Indian population evaluating the acetabular morphometry of 104 individuals, and studies by Baharuddin and colleagues and Zeng and colleagues evaluating Malaysian and Chinese populations, respectively^2,21^.

Our study measured several morphometric parameters including the CE angle, acetabular angle, acetabular version, acetabular depth, and joint space width at three different levels. Similar studies by Saikia and colleagues and Baharuddin and colleagues measured comparable parameters, with slight variations^16,21^. The CE angle, introduced by Wiberg in 1939, normally ranges from 25 to 45°, with angles below 20° indicating dysplasia^22^; in our study, the mean CE angle was 45.07±4.9°, with no cases of dysplastic hips identified.

Similarly, the mean values of the CE angle in males and females on the right and left sides did not show any statistically significant difference (*P*=0.23 on the right side, *P*=0.69 on the left side). Baharuddin *et al.* ^21^ reported an insignificant difference in the mean CE angle between the right and left sides in males (*P*=0.94) and females (*P*=0.10). In our study, CE angle did not show any significant correlation with age and laterality^22^.

In our study, the CE angle showed a statistically significant correlation in males with age, but it was insignificant in females^22^. The CE angle showed a significant correlation with BMI on both the right and left sides, and in males but not in females^24^. The mean acetabular angle, frequently used to determine dysplasia, showed no cases of dysplastic hip in our study^25^.

The acetabular angle in males and females and between the right and left sides did not show any significant differences^7,21^. The acetabular angle also showed no significant correlation with age but showed a significant correlation with BMI and laterality^7^. Detailed studies on acetabular version have reported normal mean values, with our study showing slightly higher values compared to previous studies^16,17,26^.

In our study, acetabular version did not show a significant correlation with age, BMI, or laterality, similar to the findings by Zeng *et al.* ^2^. The mean acetabular depth, considered important for defining acetabular dysplasia, was within the normal range and did not differ significantly between males and females^6,17,28,29^.

JSW measurements revealed higher values at the inferomedial site compared to the apex or superolateral site, contrasting with Lequesne *et al.*‘s^28^ findings. Our study showed no significant difference in JSW between males and females, or between the right and left sides^16,28^. There was no significant correlation of JSW with age or BMI, consistent with Goker *et al.*‘s^29^ study.

In our study, no significant correlation of JSW with age, gender, or BMI was observed, aligning with findings by Park *et al.* and Goker *et al.* ^22,29^. Similar to previous studies, our results showed no significant differences in acetabular depth and acetabular version between right and left sides or between genders^16,21^. This highlights the consistency of these morphometric parameters across different populations.

Correlation analyses in our study revealed no significant relationships between acetabular parameters and age, gender, or BMI, except for a weak positive correlation between CE angle and BMI, particularly in males. These findings indicate that while some parameters may show slight variations with demographic factors, the overall acetabular morphology remains relatively stable across different populations.

### Limitations of the study

One significant limitation of our study is the use of purposive sampling, which may introduce selection bias and limit the generalizability of our findings by over-representing certain demographics and under-representing others. Additionally, the cross-sectional nature of our study prevents us from observing changes over time or establishing causality, and the detailed anatomical measurements obtained through CT scans, while precise, may not be easily applicable to broader populations with varying access to advanced imaging technologies. These limitations highlight the need for caution when extrapolating our results and underscore the importance of conducting further studies with more representative sampling methods and longitudinal follow-ups.

## Conclusion

The mean CE angle, acetabular angle, acetabular version, acetabular depth, joint space width at SM level, joint space width at AS level and joint space width at SL level in our study were 45.07±4.92°, 35.12 ±4.17°, 22.00±3.48°, 18.00 ±2.07 mm, 7.04 ±1.22 mm, 6.70 ±1.05 mm and 5.20±1.17 mm, respectively. There was no significant difference in the means of these parameters based on gender and laterality. The CE angle and acetabular depth in male showed a significant correlation with age. Similarly, CE angle and acetabular angle showed significant correlation with BMI. Likewise, the CE angle and acetabular angle on both sides showed significant correlation with BMI. The CE angle and acetabular angle in male also showed a significant correlation with BMI.

## Ethical approval

The ethical approval for this study (Ref no: 317/076/077-Institutional Review Committee) was provided by. the Institutional Review Committee of B.P. Koirala Institute of Health Sciences, Nepal on 5 March, 2020.

## Consent

Informed consent was obtained.

## Source of funding

None

## Author contribution

B.A., S.P., M.B., K.A. and M.K.G. conceived and designed the study, with B.A., acting as the guarantor. B.A. and P.A. performed the literature review. B.A. and P.A. contributed to data collection and acquisition; performed the data, and statistical analysis. B.A. and P.A. wrote the first draft of this manuscript. B.A. and P.A. contributed to final reviewing and editing of the manuscript before submission to the journal. B.A., S.P., and M.K.G. contributed to the critical revisions before approving the final draft of the manuscript for publication.

## Conflicts of interest disclosure

The authors declare that they have no financial conflict of interest with regard to the content of this report.

## Research registration unique identifying number (UIN)

This is a cross-sectional involving a human subject, so registration of research study was done.

1. Registry used: Researchregistry.com
2. Unique Identifying number or registration ID: researchregistry10182
3. https://www.researchregistry.com/browse-the-registry#home/

## Guarantor

Bijay Adhikari is the guarantor of the study.

## Data availability statement

The datasets supporting the conclusions of this article are included within the article.

## Provenance and peer review

Not commissioned or externally peer-reviewed.
